# Visceral Varicella-Zoster Virus Infection Presenting with Severe Abdominal Pain without a Rash in a Patient with Psoriatic Arthritis Treated with Infliximab Biosimilar and Steroids: A Case Report

**DOI:** 10.31138/mjr.32.3.280

**Published:** 2021-06-25

**Authors:** Nikolaos Spernovasilis, Maria Raissaki, Ioanna Papakitsou, Sofia Pitsigavdaki, Kypros Louka, Emmanouil Tavlas, Diamantis P. Kofteridis

**Affiliations:** 1 Department of Internal Medicine and Infectious Diseases, University Hospital of Heraklion, Heraklion, Greece; 2 Department of Radiology, University Hospital of Heraklion, Heraklion, Greece; 3 Department of Rheumatology, University Hospital of Heraklion, Heraklion, Greece; 4 Department of Vascular Surgery, University Hospital of Heraklion, Heraklion, Greece

**Keywords:** Visceral varicella zoster, immunosuppression, abdominal pain, periarterial fat stranding, computed tomography

## Abstract

Visceral herpes zoster following reactivation of dormant varicella-zoster virus can rarely occur, usually in highly immunosuppressed patients, and may present with abdominal pain without the relevant rash. In the absence of skin manifestations, diagnosis of visceral herpes zoster is extremely difficult, while computed tomography may reveal isolated periarterial fat stranding. We describe a rare case of visceral herpes zoster in a medically immunocompromised adult with psoriatic arthritis, who presented with acute abdomen, was diagnosed based on computed tomography findings and subsequent serum polymerase chain reaction results, and was appropriately treated with an uneventful recovery. This case underlines the significance of considering varicella-zoster virus infection as a cause of severe abdominal pain even in the absence of rash in this setting, and highlights the potential role of appropriately performed computed tomography in such unusual and complex cases, where early diagnosis and initiation of treatment is extremely important for a favorable outcome.

## INTRODUCTION

Reactivation of dormant varicella-zoster virus (VZV) results in herpes zoster (HZ), which occurs more frequently in older or immunocompromised individuals.^1^ HZ usually presents as a unilateral, painful dermatomal rash. However, visceral involvement can rarely occur, principally in highly immunosuppressed patients.^1^ In the absence of skin eruption, diagnosis of visceral HZ is challenging. Severe abdominal pain as first clinical symptom may erroneously lead to the diagnosis of acute abdomen and to unnecessary exploratory laparotomy,^2^ posing a significant risk for delayed treatment and unfavorable outcome. In addition, few cases have reported perivascular fat stranding on computed tomography (CT) in the setting of visceral HZ.^3,4^

This report details a rare case of visceral HZ with abdominal pain but no skin manifestations at diagnosis in a patient with psoriatic arthritis who was being treated with infliximab biosimilar (Inflectra®) and had recently received high doses of steroids. This case also underscores the potential value of imaging in evoking the suspicion of visceral HZ and triggering appropriate investigations for definitive diagnosis and prompt effective treatment.

## CASE PRESENTATION

A 44-year-old woman presented to another hospital with a few hours’ history of severe epigastric pain radiating to the back, accompanied by nausea. Her past medical history was significant for psoriatic arthritis treated with infliximab biosimilar (Inflectra®) for the past two years. The last infliximab biosimilar (Inflectra®) infusion had been 15 days prior to presentation. At that time, due to a severe arthritis flare, the patient also received a single dose 500 mg of IV methylprednisolone, with instructions to complete a 10-day course of oral prednisolone, 30 mg per day with gradual decrease of the dose.

**Figure 1.**
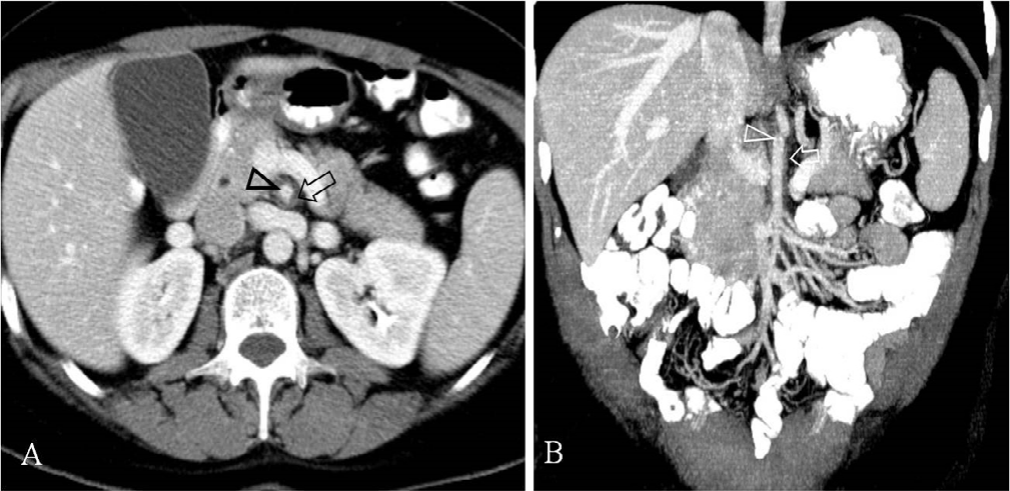
CT scan following intravenous contrast administration, split-bolus excretory-portal phase. (A) Axial slice shows the superior mesenteric artery containing a hypoenhancing line (arrowhead) and surrounded by perivascular fat stranding (arrow). (B) Coronal reconstruction shows a short line at the upper aspect of the lumen (arrowhead) and thickening of the adjacent wall (arrow). Findings were initially interpreted as dissection.

Her vital signs were normal. Abdominal examination revealed tenderness to deep palpation in the epigastrium, without guarding or rebound. The remainder of the clinical examination, including skin and nervous system, was unremarkable. Laboratory tests showed lymphopenia (1,000/μL) and mild anemia (13.6 g/dL). The rest of the blood tests, including liver enzymes and serum and urine amylase, were within normal range. A chest radiograph was normal. Abdominal computed tomography (CT) reported periarterial fat stranding and possible dissection of the superior mesenteric artery (SMA) (**Figure 1**) and the patient was transferred to our hospital for further evaluation and treatment.

**Figure 2.**
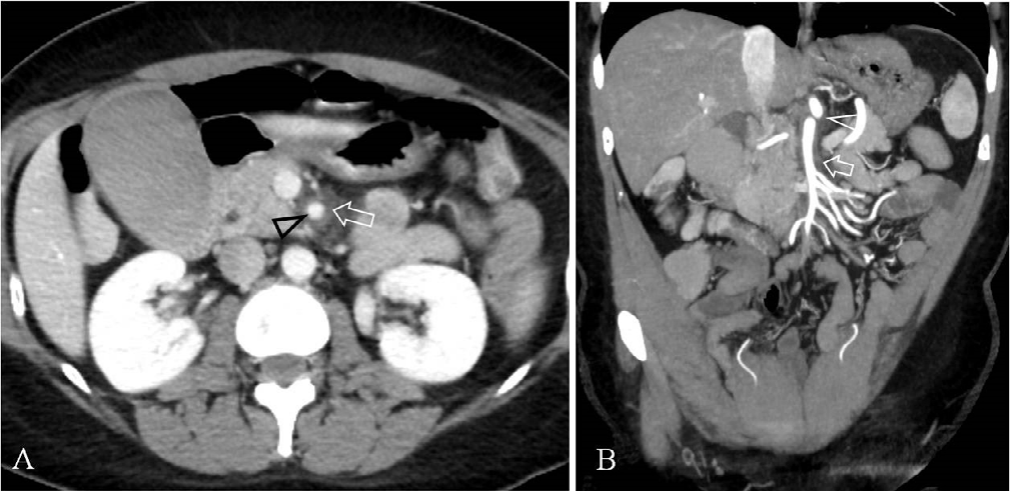
CT scan following intravenous contrast administration, portal phase. (A) Axial slice shows the enhancing superior mesenteric artery (arrowhead) with an intact smooth lumen, without evidence of dissection. Note perivascular fat stranding as circumferential increased density of adjacent fat (arrow). (B) Maximum intensity projection reconstruction in the coronal plane shows the extent of perivascular fat stranding (arrow) which was appreciated as progressive compared to the initial scan and situated around the normal lumen of superior mesenteric artery and adjacent celiac artery (arrowhead).

**Figure 3.**
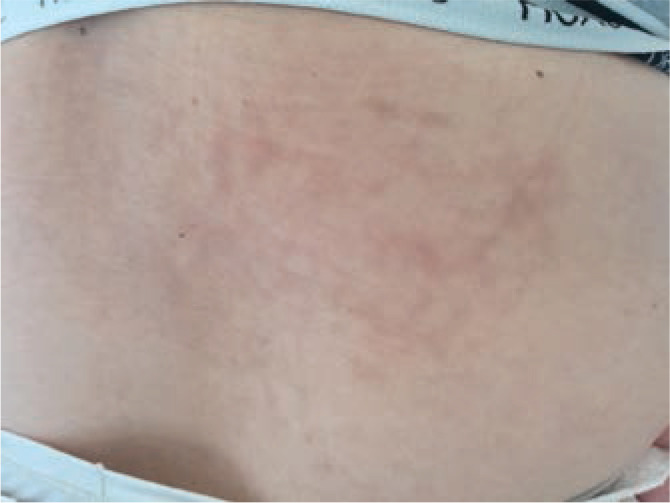
A non-pruritic macular rash emerged on the patient’s back on the second day of hospitalization, without further evolution.

On presentation, CT images were reviewed, findings were considered equivocal, and a new contrast-enhanced abdominal CT scan with an angiographic arterial phase was performed. Perivascular fat stranding was noted as the single abnormal finding while the arteries were found patent without evidence of dissection (**Figure 2**). Due to isolated periarterial fat stranding, the possible diagnosis of visceral VZ was raised and the patient was admitted to hospital for further evaluation. On a subsequent further review of patient’s medical history, it was found that she had been diagnosed with varicella during her childhood. On the second day of hospitalization, a skin eruption without blistering emerged on the patient’s back (**Figure 3**). Varicella-zoster virus DNA was detected by polymerase chain reaction (PCR) in the serum and the diagnosis of visceral HZ was established. The patient received 4 days of intravenous acyclovir and was discharged with an additional 10-day course of oral valacyclovir. On her follow-up visits, two and ten weeks later, the pain and the rash had subsided, and the patient was in a good health condition in general.

## DISCUSSION

Visceral VZV infection is an uncommon emergency condition, associated with increased mortality.^5,6^ It usually occurs due to reactivation of dormant VZV, presenting as HZ, in immunodeficient or highly immunosuppressed individuals with impaired cell-mediated immunity.^1,5^ Most cases have been described in hematological patients, mainly among individuals with a history of hematopoietic stem-cell transplantation.^5,6^ There have also been rare cases of visceral involvement in patients with autoimmune or inflammatory bowel diseases attributed to the use of immunosuppressive therapy, such as steroids, disease-modifying antirheumatic drugs, biological agents, and small-molecule drugs.^6,7^ Among these drug classes, steroids, either as monotherapy or as part of combined immunosuppressive therapy, display the strongest correlation with VZV reactivation overall.^7,8^ Similarly, in our patient with psoriatic arthritis, the temporal association between the addition of high-dose of steroids on top of infliximab biosimilar (Inflectra®) and the occurrence of visceral HZ implies a causal relation.

Most patients with visceral HZ reported in the literature present with severe abdominal pain as the cardinal symptom.^2,3,5,6,9^ A significant proportion also experiences one or more of the following: nausea, vomiting, diarrhea, mental status disturbances, radiographic evidence suggesting pneumonitis, and elevated liver enzyme or amylase levels.^2,5,6,9^ VZV-associated gastritis, intestinal mucosal necrosis and hemorrhage, stretching of the hepatic capsule due to VZV-hepatitis, VZV-induced pancreatitis, VZV invasion into celiac ganglia, VZV neuropathy of the parietal peritoneum, and periarterial fat stranding around the main intra-abdominal arteries, have been proposed as possible causes for the abdominal pain.^3,5,6,9^ In many patients, abdominal pain precedes the characteristic vesicular rash by a few days, while some either develop atypical cutaneous eruptions, as our patient did, or never experience a rash.^5,9^ This may cause significant diagnostic problem and may even lead to unnecessary surgical interventions.^2^ Hence, it is imperative that medical professionals caring for the aforementioned groups of patients consider visceral HZ in otherwise unexplained abdominal pain and perform an appropriate work-up, since delays in the institution of adequate antiviral treatment have been proven life-threatening.^6^

Herpes zoster in immunocompetent individuals is usually diagnosed clinically, based on the characteristic vesicular skin eruption with dermatomal distribution.^1^ However, in severely immunocompromised patients in whom atypical skin manifestations or isolated visceral involvement may occur, laboratory confirmation is needed.^1^ The diagnostic technique of choice is PCR, because of its sensitivity (over 90% in different clinical specimens), specificity, and short turnaround time for results.^1,6,10^ The PCR assays of the whole blood, plasma or serum enable the prompt diagnosis of visceral HZ in the absence of rash, as in the case described here.^1^ There are also available PCR assays for cutaneous, cerebrospinal fluid, bronchoalveolar lavage, and vitreous humor specimens.^1,5^ Other diagnostic techniques, with significantly lower sensitivity than PCR, include direct fluorescent antibody assays on tissues or secretions, and viral culture.^1,5^ Serological tests are of limited value for the diagnosis of HZ since IgM antibodies are detectable in only 50%–60% of patients with HZ, irrespective of their immune status. ^10^

Computed tomography has been increasingly performed in patients with acute persisting abdominal pain unexplained by clinical, laboratory tests, and ultrasonography. Periarterial fat stranding may be encountered in several conditions (**Table 1**). In our patient, periarterial fat stranding was an isolated finding, involving both the SMA and the celiac artery. This setting is extremely unusual in routine emergency radiology and favoured the diagnosis of visceral HZ.^3,4^ In immunocompromised patients, isolated periarterial vascular stranding can precede skin manifestations HZ by many days and should be considered highly suggestive of visceral HZ until proven otherwise.^3,4^ Novel therapeutic approaches for many diseases involve extensive immunosuppression. This has led to a notable expansion of immunocompromised populations, which are at high risk for uncommon infections or unusual presentation of common infections. The present rare case of visceral HZ in a medically immunocompromised individual with an autoimmune disease underlines the significance of considering VZV infection as cause of severe abdominal pain in these patients, even in the absence of rash. It also highlights the potential role of appropriately performed imaging and correct interpretation of isolated periarterial fat stranding in the investigation of such unusual and complex cases. Similar cases may be increasingly common in the future, making early diagnosis and initiation of treatment of paramount importance.

**Table 1.** Differential diagnosis of conditions that may cause fat stranding around the superior mesentery artery and the celiac artery.

**Entity**	**Periarterial fat stranding**	**Vessel wall abnormality**	**Vessel luminal abnormalities**	**Additional findings**
Vasculitis	Occasionally present	Wall thickening	Beading of the lumen (alternating narrowing and normal-caliber lumen)	Evidence of bowel ischemia with bowel wall thickening, ascites, wall hypoenhancement or hyperenhancement depending on the stage of bowel ischemia
Pancreatic cancer	Occasionally present	Wall thickening with obliteration of fat planes between pancreatic lesion and SMA	Luminal narrowing due to compression or infiltration	Focal lesions in the pancreatic gland with different degree of contrast enhancement compared to the remaining pancreas, lymphadenopathy
Pancreatitis	Present, associated with peripancreatic fat stranding and thickening of retroperitoneal fasciae	Absent wall thickening	Rarely pseudoaneurysm formation or venous thrombosis	Pancreas enlargement, occasionally hypoenhancing necrotic parenchyma, pseudocyst formation
Aortic aneurysm rupture	Present, associated with retroperitoneal hematoma and thickening of retroperitoneal fasciae	Wall thickening, atherosclerotic changes of aorta	Luminal dilatation of aorta	Hypoenhancement due to infarcts in abdominal organs, thin IVC in hypovolemic shock
Aortic aneurysm dissection	Present in impeding or occurred rupture, associated with retroperitoneal hematoma	Absent wall thickening	Visible intimal flap, intraluminal thrombus or differential enhancement of true and false lumen in aorta, luminal dilatation	Dissection may extend into other vessels
SMA dissection	Minimal when present	Absent wall thickening	Visible intimal flap in SMA	No additional findings
Retroperitoneal or mesenteric trauma	Present	Absent wall thickening	Absent luminal dilatation. Posttraumatic vasospasm possible.	Contrast extravasation in vascular injuries, traumatic findings in solid abdominal organs, mesenteric and bowel wall anomalies in bowel trauma, hemoperitoneum, history of trauma
VZV vasculitis	Present	Absent or circumferential wall thickening	Absent	Absent

SMA: superior mesenteric artery; IVC: inferior vena cava; VZV: Varicella zoster virus.
